# Interdisciplinary collaborative skill development in a health research training program in Zambia

**DOI:** 10.1186/s12909-026-08867-8

**Published:** 2026-02-21

**Authors:** Julie M. Buser, Maureen M. Masumo, Ella August, Swebby Macha, Rachel Gray, Bellington Vwalika, Tamrat Endale, Alice Ngoma-Hazemba, Yolanda R. Smith

**Affiliations:** 1 Center for International Reproductive Health Training (CIRHT), University of Michigan, North Ingalls Building Suite 947, Ann Arbor, Michigan 48105 USA; 2 Department of Midwifery, Women’s and Child Health, School of Nursing Sciences, University of Zambia, Lusaka, Zambia; 3 Department of Epidemiology, University of Michigan School of Public Health Editor-in-Chief, PREPSS (Pre-Publication Support Service), Ann Arbor, Michigan USA; 4 Department of Obstetrics & Gynecology, University of Zambia, Lusaka, Zambia; 5 Department of Obstetrics & Gynecology School of Medicine, University of Zambia, Lusaka, Zambia; 6 Department of Community and Family Medicine, School of Public Health, University of Zambia, Lusaka, Zambia; 7 Department of Obstetrics and Gynecology, Center for International Reproductive Health Training (CIRHT), University of Michigan, Ann Arbor, Michigan USA

**Keywords:** Collaborative skills, Research capacity-strengthening, Interdisciplinary collaboration, Sexual and reproductive health, Health professionals, Zambia, Communication, Leadership, Institutional barriers, Mentorship

## Abstract

**Background:**

The increasing complexity of sexual and reproductive health research is strengthened by interdisciplinary collaboration. While technical proficiency receives significant attention in research capacity-strengthening, the development of core collaborative skills, such as communication, teamwork, conflict resolution, and leadership, remains underemphasized. The Center for International Reproductive Health Training at the University of Michigan (CIRHT-UM) integrated collaborative skills training into a comprehensive research-capacity strengthening program for health professionals in Zambia, combining technical research instruction with explicit interpersonal skill development. This study explored CIRHT-UM participants’ experiences, perceptions, and outcomes related to collaborative research skills developed during a health research capacity strengthening program in Zambia.

**Methods:**

Data were collected in June 2025, following completion of the training and seed grant activities, to explore participants’ experiences, perceptions, and outcomes related to collaborative skill development during the program, using a qualitative phenomenological approach, through nine in-depth interviews and three focus group discussions with 28 Zambian professionals, including lecturers, nurses, midwives, physicians, and public health practitioners, who received CIRHT-UM seed grants. Thematic analysis, informed by Bronfenbrenner’s *Ecological Systems Theory* and *Gearing Up* frameworks, enabled exploration of ecological influences and readiness factors in collaborative skill development to capture barriers, enablers, application, and impacts of training.

**Results:**

Thematic analysis generated four key themes: (1) expansion of professional networks and interdisciplinary exposure; (2) barriers created by hierarchies, siloed work, and inequities; (3) growth in communication, leadership, and problem-solving skills; and (4) influence of environmental constraints and systemic supports on collaboration. Participants reported increased confidence and teamwork abilities, but highlighted ongoing challenges from heavy workloads and limited institutional support. Focus groups emphasized organizational barriers, while interviews provided deeper insights into personal growth and readiness for collaboration.

**Conclusions:**

Embedding collaborative skill development within a research training program in Zambia enhances both personal growth and research quality. Egalitarian team environments, structured mentorship, and peer learning were identified as critical facilitators, while entrenched hierarchies and lack of protected time remained significant barriers in Zambian academic and clinical environments. Purposefully designed, inclusive, and well-supported collaboration initiatives improve team effectiveness and professional satisfaction. Sustained institutional policies, research training, and adaptive team structures are essential for lasting interdisciplinary engagement. While these strategies may be relevant to other low-resource or sub-Saharan African contexts facing similar barriers, their impact is closely tied to specific local conditions and institutional structures. Future adaptation should be tailored to each country’s unique setting, workforce, and policy landscape for research training programs that intentionally cultivate collaboration to drive innovation and improve health outcomes.

**Supplementary Information:**

The online version contains supplementary material available at 10.1186/s12909-026-08867-8.

## Background

The growing emphasis on interdisciplinary research and global collaboration highlights the necessity of developing collaborative skills as a core component of successful research practice [1, 2]. Research teams increasingly consist of members from various disciplines, institutions, and cultures. Consequently, effective collaboration, communication, teamwork, and leadership are essential for achieving shared goals [3, 4]. Despite the importance of interpersonal skills in effective collaborations, traditional research training programs prioritize technical and methodological expertise [5]. Addressing this gap, the Center for International Reproductive Health Training at the University of Michigan (CIRHT-UM) integrates collaborative skills training into its curriculum, strengthening the teamwork skills among researchers in Sub-Saharan Africa within community and clinical settings [6]. The CIRHT-UM program was designed primarily to build technical research competencies among health professionals while deliberately integrating collaborative skills training such as communication, teamwork, conflict resolution, and leadership as essential components.

The need for collaborative skills is particularly acute in complex research projects that necessitate integrating diverse expertise to tackle multifaceted sexual and reproductive health and rights (SRHR) issues due to the field’s sensitivity, intersectionality, and the need to navigate ethical, legal, and cultural complexities [7]. Skills such as conflict resolution, trust-building, and leadership directly influence researchers’ productivity primarily in collaborative skill development training environments, research quality [8], and ability to secure funding, ultimately driving scientific innovation [9, 10]. Collaborative skills, including effective communication, teamwork, conflict resolution, and leadership, are essential for all researchers conducting health research, not only in local or specialized contexts but globally. Evidence from multiple studies across international settings demonstrates that research teams with strong collaborative competencies consistently achieve higher productivity, innovation, and research quality, regardless of discipline or geography [3, 4, 8, 9]. These collaborative skills form a universal foundation for successful health research teams worldwide. Researchers’ backgrounds, including their disciplinary expertise, professional experiences, and cultural perspectives, can significantly influence how research questions are framed, data are collected, and findings are interpreted [11–13] In studies that emphasize interdisciplinary collaboration, recognizing and reflecting on this diversity is crucial for minimizing bias and enhancing the depth and validity of qualitative analysis [14–16]. However, the specific mechanisms for effectively developing these skills within SRHR training programs remain largely unexplored, illuminating a critical gap in understanding participant experiences and perceptions in structured training environments.

To address this gap, our study examined how a training program designed to foster personal growth and systematic support facilitates team-based research practices. Our intervention was designed to incorporate two complementary theoretical frameworks: Bronfenbrenner’s *Ecological Systems Theory* and the *Gearing Up* conceptual framework by Mulvale and colleagues [17–19]. The *Ecological Systems Theory* offers a detailed model for understanding how various environmental systems, from immediate social interactions to broader institutional and societal contexts, shape individual development [18]. Bronfenbrenner’s framework guides our examination of how individual, interpersonal, organizational, and broader contextual factors affect the development and application of collaborative skills. Meanwhile, the *Gearing Up* framework emphasizes the importance of readiness and capacity for effective interdisciplinary collaboration at the individual and organizational levels [19]. The *Gearing Up* framework enables us to assess the levels of readiness and capacity that make skill uptake and collaboration possible. These models offered a comprehensive lens to study the factors that influence collaborative skill development.

Our study investigated the CIRHT-UM program participants’ experiences, perceptions, and outcomes regarding collaborative skills development within the context of a health research capacity strengthening program at the University of Zambia (UNZA). This program has interdisciplinary collaborative skills training embedded within the trainings, workshops and seed grant program. For our first aim, we explored how participants’ experiences and perceptions of collaborative skill development were influenced by ecological factors such as team dynamics and institutional culture, alongside the assessment of readiness to develop skills like communication, teamwork, conflict resolution, and leadership. The second aim focused on how these collaborative skills are put into practice, considering the settings in which they are enacted, participants’ and institutions’ preparedness, and the challenges, strategies, and gaps that emerge during implementation. By analyzing both environmental influences and readiness factors, this study addresses the central research question: What are the experiences, perceptions, and outcomes related to collaborative skills development from a health research capacity strengthening program in Zambia? These insights contribute to the literature and inform the design of research training programs that better prepare global scientists for the collaborative demands of contemporary health research.

## Methods

### Research training program description

In a collaborative partnership with UNZA, CIRHT-UM delivered a comprehensive research training program designed to enhance the research skills in reproductive health and collaborative skills such as communication, teamwork, and leadership. Participants included Zambian faculty, lecturers, nurses, midwives, physicians, and public health professionals who received CIRHT-UM seed grants. The program targeted participants with a range of skills and research experience and employed a blend of remote webinars and in-person trainings. Notably, all participants received a seed research grant from CIRHT-UM, and forming an interdisciplinary team was a requirement to be eligible for this grant. Participants were either assigned or required to identify mentors to support them throughout the research process. The training program curriculum was informed by a comprehensive survey of researchers’ comfort and proficiency across key areas of the research process, including literature review, mentoring, research design, protocol development, interdisciplinary collaboration, data management and analysis, publication, and grant writing. This gap analysis enabled us to identify strengths and areas needing additional support, allowing for the design of targeted training activities. The CIRHT-UM research training program consisted of foundational technical research training modules, supplemented by targeted collaborative and interpersonal skills workshops, to ensure participants developed both methodological expertise and essential teamwork competencies.

After completion of the initial webinars and in-person training on proposal writing, the seed grant recipients were selected. Next, a two-day in-person training was provided to the seed grant PIs giving an overview of principal investigator (PI) responsibilities. The training emphasized the importance of collaboration in the PI role. The session began with a concise overview that highlighted how teamwork was crucial across all aspects of managing research projects, from administration and grant management to resource planning and delegation. This introduction set the tone for participants to reflect on their current practices and recognize areas where collaboration could be strengthened. During the training, participants completed exercises on conflict management that encouraged participants to work together to develop solutions for issues like distributing responsibilities and addressing resource constraints. After group discussions, participants reconvened to share their strategies, allowing everyone to benefit from diverse perspectives and approaches. To conclude, participants developed personalized action plans outlining steps and communication methods to enhance collaboration within their own teams and mentors. The training provided attendees with practical tools and new strategies, empowering them to build more effective and cohesive research environments.

**Fig. 1:**
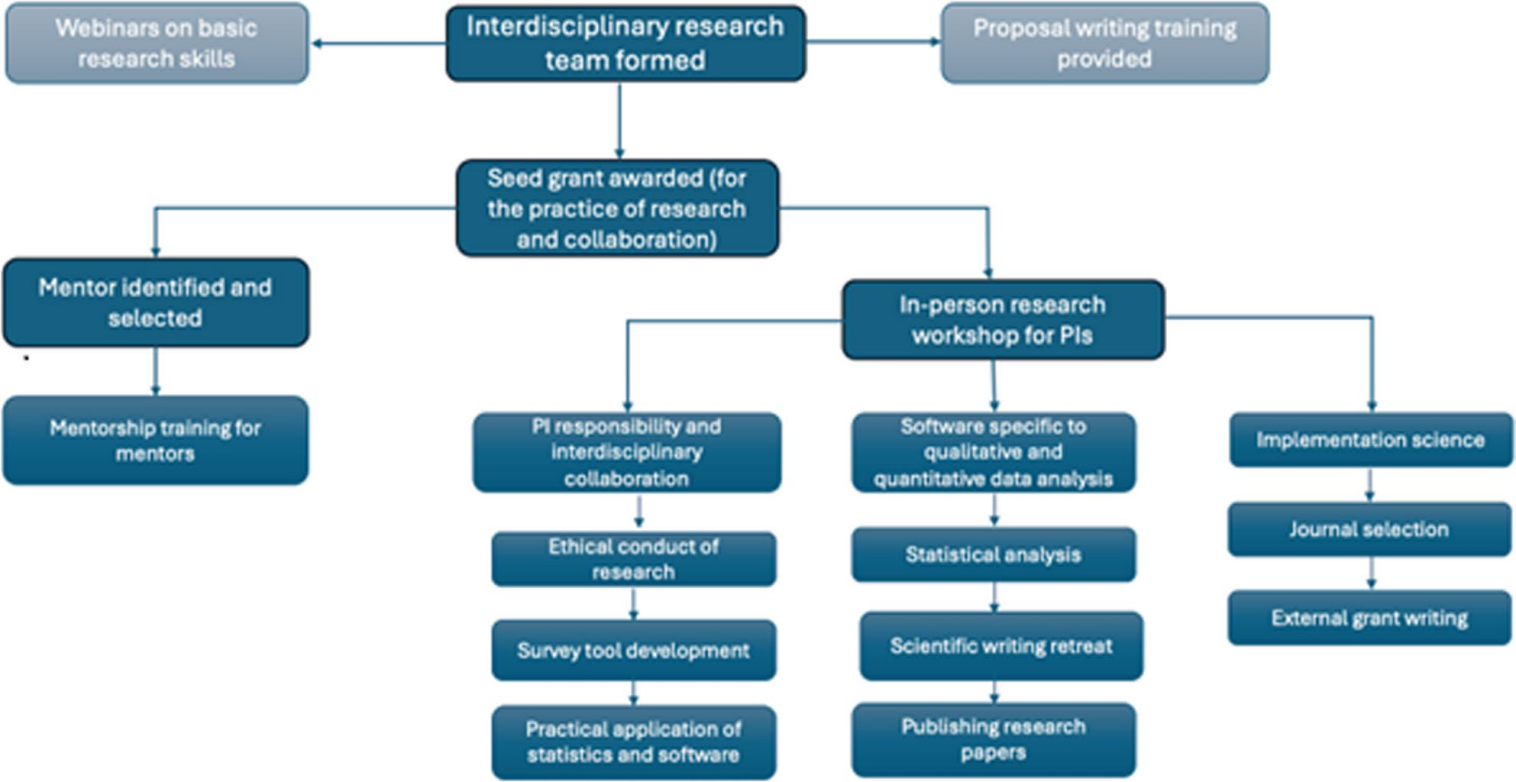
Description of the Center for International Reproductive Health Training research training program

In-person sessions trained participants on ethical conduct of research, survey tool development, and the practical application of statistics and software. Participants also received training in statistical analysis, as well as software specific to qualitative and quantitative data analysis. Additional workshops and trainings focused on scientific writing, publishing research papers [20], journal selection, implementation science [21], and external grant writing. The mentors also received a one-day in-person training on mentorship [22]. Together, these modules equipped participants and their teams with the knowledge and practical skills needed to conduct robust, ethical, and impactful research in reproductive health. Figure 1 provides a summary description of the CIRHT-UM research training program.

### Research team and reflexivity

Interviews were audio-recorded and transcribed verbatim by three Zambian research assistants with extensive experience conducting qualitative research. The interviewing team comprised one development studies practitioner with a master’s in public health (MPH) in health policy, one physiotherapist with an MPH in epidemiology, and one medical doctor with an MPH in health promotion. None of the research assistants had any affiliation with the CIRHT-UM research program prior to study commencement.

Throughout the study, all members of the research team engaged in continuous reflexivity to recognize and address potential biases and the influence of our own perspectives on data collection, analysis, and interpretation. By openly discussing our diverse disciplinary backgrounds and prior experiences with collaborative research, we aimed to minimize the impact of preconceived notions and ensure that participants’ voices and experiences remained central throughout the research process.

### Theoretical frameworks

This study was guided by Bronfenbrenner’s *Ecological Systems Theory* and the *Gearing Up* framework. Bronfenbrenner’s *Ecological Systems Theory* posits that human development is shaped by the nested interactions of individuals with their immediate settings (microsystem), relationships between those settings (mesosystem), surrounding institutions (exosystem), broader societal influences (macrosystem), and the dimension of time (chronosystem) [23–25]. The *Gearing Up* framework is a model that identifies and evaluates key dimensions of readiness, such as motivation, capacities, and resources, at both individual and organizational levels, enabling systematic assessment and strengthening of preparedness for collaboration [19]. Bronfenbrenner’s model helped elucidate the multiple layers of influence on collaborative skills development, from individual interactions to societal norms, by examining how these systems impact the training and application of such skills. The *Gearing Up* framework helped us assess readiness and capability, both in individuals and organizational structures, providing a lens to evaluate preparedness for effective collaboration. Integrating elements from both, we explored individual knowledge and systemic readiness for collaboration, capturing data on environmental influences and structural capacities.

### Participant selection

The participants were all seed grant awardee faculty members affiliated with UNZA, or the University Teaching Hospital in Lusaka, and were selected for their roles as CIRHT-UM seed grant awardees. They were asked to join the study via email and WhatsApp with an invitation and follow-up phone calls. Written informed consent was obtained from all participants prior to data collection. Prospective participants received a detailed information sheet via email and WhatsApp that explained the study’s purpose, procedures, risks, and benefits. No incentives were provided for participation. Before each interview or focus group discussion commenced, the consent process was revisited, and participants signed or digitally confirmed informed consent forms indicating their voluntary agreement to participate. They were assured that their participation was voluntary, that they could withdraw at any time without penalty, and that their responses would be anonymized and remain confidential. Twenty-eight of the 35 recipients of seed grants from the CIRHT-UM program joined the participant group. No seed grant recipient declined to participate, however, seven were unavailable for interviews due to scheduling conflicts or membership on the study/author team. Seed grant recipients were selected as participants because they directly experienced the full CIRHT-UM training program, which included both technical and collaborative skill development modules, and actively participated in interdisciplinary team research. Focusing exclusively on these individuals ensured that all study participants had comparable exposure to the intervention and could provide relevant insights into the impact of collaborative skills training. Unsuccessful applicants or broader stakeholders were excluded to maintain consistency in the intervention experience and optimally address our research question regarding collaborative skill development. Participants included trainees and faculty of obstetrics & gynecology, nursing, and midwifery programs, along with public health professionals at the University of Zambia and the University Teaching Hospital in Lusaka. The variety in professional backgrounds allowed for comprehensive insights into collaborative skills dynamics. Participants in focus groups were intentionally selected to include a diverse range of professionals.

### Setting

All focus groups were conducted at a hotel conference center in Lusaka, Zambia, offering a neutral and accessible environment for group interaction. In-depth interviews (IDIs) took place at various locations, including the UNZA- Ridgeway campus, University Teaching Hospital, or Chilenje Level 1 Hospital in Lusaka, Zambia. Three IDIs were done virtually via Google Meet. No one else was present during focus group discussions (FGDs) or IDIs other than the participants and research assistants. FGDs and IDIs were conducted separately and served as distinct qualitative data collection methods, allowing us to capture both collective group dynamics (via FGDs) and individual experiences (via IDIs) among CIRHT-UM seed grant participants. The research program training sessions were conducted at various hotels and conference centers in Lusaka.

### Data collection

In-depth, semi-structured interviews were conducted in June 2025 face-to-face using open-ended questions to allow participants to describe their experiences of collaborative skills development while conducting research, in their own words. Appendix 1 shows the questions from the interview guide. The development of the data collection tool was informed by a variety of resources, such as the foundational materials included internal program documentation, training syllabi, and evaluation frameworks, which provided context-specific starting points for the interview guide. Foundational materials included internal program documentation, training syllabi, and evaluation frameworks, which provided context-specific starting points for the interview guide [26]. In addition, the *WHO Framework for Action on Interprofessional Education & Collaborative Practice* [27] was consulted to ensure alignment with international best practices for fostering collaboration across health professions. The landmark National Academies volume, *Facilitating Interdisciplinary Research* [28], offered key guidance on overcoming barriers and establishing effective interdisciplinary teams, while the *Collaboration and Team Science: A Field Guide* from the NIH, served as a practical manual for operationalizing collaborative research and team dynamics in academic and clinical settings [29]. Together, these sources shaped a rigorous, evidence-based approach to developing the interview guide.

We combined IDIs and FGDs to comprehensively explore collaborative skill development from both individual and group perspectives. FGDs were suited to eliciting collective experiences, team dynamics, and organizational influences, highlighting how participants navigated collaboration within institutional contexts. In contrast, IDIs provided a confidential space for participants to reflect on their personal growth, motivations, and unique strategies, enabling us to capture nuanced insights that may not emerge in group settings. This integrated approach allowed us to triangulate findings and better understand both shared and individual pathways to collaborative competency.

Probing questions were included to elicit rich, detailed descriptions of participants’ perceptions and outcomes related to collaborative skills development during a health research capacity strengthening program in Zambia. The interview guide was pilot tested on three faculty members at UNZA. Each research assistant conducted three IDIs and one FGD.

The nine IDIs lasted, on average, 35 min (range: 26 to 58 min). The three FGDs lasted, on average, 104 min (range: 94 to 117 min), with 6–7 members in each for a total of 19 participants. All interviews and focus groups were conducted in English. Field notes were made during and after each interview to record contextual observations and interviewer reflections. All sessions were audio-recorded and transcribed verbatim, with transcripts checked for accuracy against the recordings.

### Ethical considerations

Our study was conducted in accordance with the Declaration of Helsinki. Institutional Review Board (IRB) approval was obtained prior to beginning the study from the University of Zambia Biomedical Research Ethics Committee (REF. NO. 5275 − 2024). The University of Michigan received an IRB exemption to conduct the study (HUM00203642). Confidentiality was maintained and privacy was respected throughout the study. Data files were stored securely on password-protected computers accessible to the research team only.

We recognize that the power differential between the research team and participants, who were recipients of seed grants, may have influenced participation and the nature of disclosures during data collection. To mitigate this, all interviews and focus group discussions were conducted by Zambian research assistants who had no prior affiliation with the CIRHT-UM program, and who were not involved in grant selection or program oversight. Emphasis was placed on voluntary participation during consent processes, with assurances that non-participation or candid disclosure would not affect current or future funding, support, or professional relationships. Confidentiality was stressed, and participants were reminded that their responses were anonymous and would not be shared with program funders or grant administrators.

### Data analysis

We undertook an iterative thematic analysis of interview transcripts, following the approach of Braun and Clarke [30]. Coding was conducted manually by two members of the research team, each with qualitative health research experience, who independently read through each transcript and generated initial codes using color highlighting and annotation on digital documents. An initial codebook was developed and refined throughout the process, with regular team discussions ensuring that inductive and deductive (framework-guided) codes were clearly defined and consistently applied. Both inductive (data-driven) and deductive (framework-guided) approaches were used, with Bronfenbrenner’s *Ecological Systems Theory* and the *Gearing Up* framework sensitizing the coding and theme development process. Coded segments and emerging themes were organized using spreadsheets and word processing software. Coders compared and discussed preliminary codes, resolving discrepancies through consensus to ensure consistency in theme development. Appendix 2 displays the code book used. Peer debriefing sessions were held among team members to critically review and challenge coding decisions, enhancing analytic rigor and consensus.

Throughout analysis, the research team engaged in ongoing reflexivity, openly discussing disciplinary backgrounds, preconceptions, and potential biases. Field notes and analytic memos were maintained to document reflections and support balanced interpretation of the data. Consistent with Braun and Clarke’s [30] recommendations, we did not seek or claim ‘data saturation’ as a methodological endpoint. Instead, our analytical process emphasized achieving adequate richness, complexity, and depth in understanding the phenomena under study. We prioritized transparency, reflexivity, and thorough representation of diverse viewpoints in our coding and theme construction. By including all available transcripts, we sought to maximize analytic richness and authenticity, ensuring that our findings reflected the breadth of participant experiences rather than the cessation of new information. Consistent with Braun and Clarke [30], themes were developed through interpretive analysis, focusing on meaning and relevance rather than occurrence or frequency. No frequency counts are reported, as prevalence is not a criterion for theme development in this approach.

Trustworthiness was further supported by member checking, where summarized versions of some interviews were provided to participants for feedback or clarification to ensure accurate representation of their experiences and perspectives. During thematic analysis, we systematically mapped each emergent theme onto specific ecological levels defined by Bronfenbrenner’s theory (e.g., microsystem, mesosystem, exosystem, macrosystem) and readiness domains highlighted in the *Gearing Up* framework (e.g., individual motivation, team capacity, organizational support). This process enabled us to interpret barriers, enablers, and impacts of collaborative skill development in relation to both the immediate environment and broader institutional context.

To enhance credibility, illustrative participant quotations were used to support key findings, with attributions by participant role and demographic group. An audit trail of analytical decisions and coding choices was maintained throughout the study. To further validate our findings and enhance trustworthiness, we conducted member checking by sharing summaries or excerpts with participants for feedback. While member checking was conducted by sharing interview summaries with participants, the feedback received affirmed the accuracy of the analysis and themes, and no substantial changes to the theme structure or interpretations were needed. No repeat interviews were conducted. In conducting our thematic analysis, we actively looked for deviant or contradictory cases that might diverge from the main themes. However, we found a high degree of consistency among participants’ experiences, with no significant outliers or opposing viewpoints identified.

We adhered to the Consolidated Criteria for Reporting Qualitative Research (COREQ) checklist [31]. The COREQ checklist guided the study’s planning, data collection, analysis, and reporting, enabling us to provide a comprehensive account of our methodology while enhancing the clarity and completeness of our qualitative research reporting.

## Results

### Demographics

Twenty-eight professionals participated, with a nearly equal distribution between males (*n* = 15) and females (*n* = 13) (Table 1). Participants ranged in age from 21 to 61 years, with an average age of 43.7 years. Most individuals (*n* = 21) held academic positions as lecturers, often combined with roles such as nurse, midwife, physician, or assistant lecturer. The primary affiliation of participants was UNZA (*n* = 27). Regarding educational background, 13 held a master’s degree (such as MPH, MSc, or Master of Nursing), 9 held a Master of Medicine (MMed), and 6 held a PhD as their highest degree. The most prevalent disciplines represented were public health (*n* = 15), nursing (*n* = 12), and medicine (*n* = 11; some individuals selected multiple disciplines). The group reflected strong backgrounds in public health and clinical practice and was anchored mainly at UNZA.

**Table 1:** Demographic and professional characteristics of 28 Zambian CIRHT-UM seed grant participants, 2025

Demographic	*n* (%)
Gender	
Male	15 (54%)
Female	13 (46%)
Job Title*	
Assistant Lecturer	3 (11%)
Lecturer	21 (75%)
Part-time Lecturer/Researcher	1 (4%)
Nurse	8 (29%)
Midwife	4 (14%)
Physician	5 (18%)
Highest Advanced Degrees	
Master’s Degree	13 (46%)
Master of Medicine (MMed)	9 (32%)
Doctor of Philosophy (PhD)	6 (21%)
Health Sciences Discipline*	
Health Promotion	6 (21%)
Implementation Science	1 (4%)
Medicine (including Obstetrics & Gynecology)	11 (39%)
Midwifery	7 (25%)
Nursing (including Public Health Nursing)	12 (43%)
Public Health	15 (54%)
Professional Affiliation*	
Ministry of Health	5 (19%)
University of Zambia	27 (100%)
University Teaching Hospital	5 (19%)
Women and Newborn Hospital	2 (7%)
Age (years)	
Under 25	1 (4%)
25–34	5 (18%)
35–44	9 (32%)
45–54	7 (25%)
55+	6 (21%)
Average	44

*Some individuals selected multiple titles, disciplines, and affiliations, making totals in those categories exceed the sample size (*n* = 28)

**Fig. 2:**
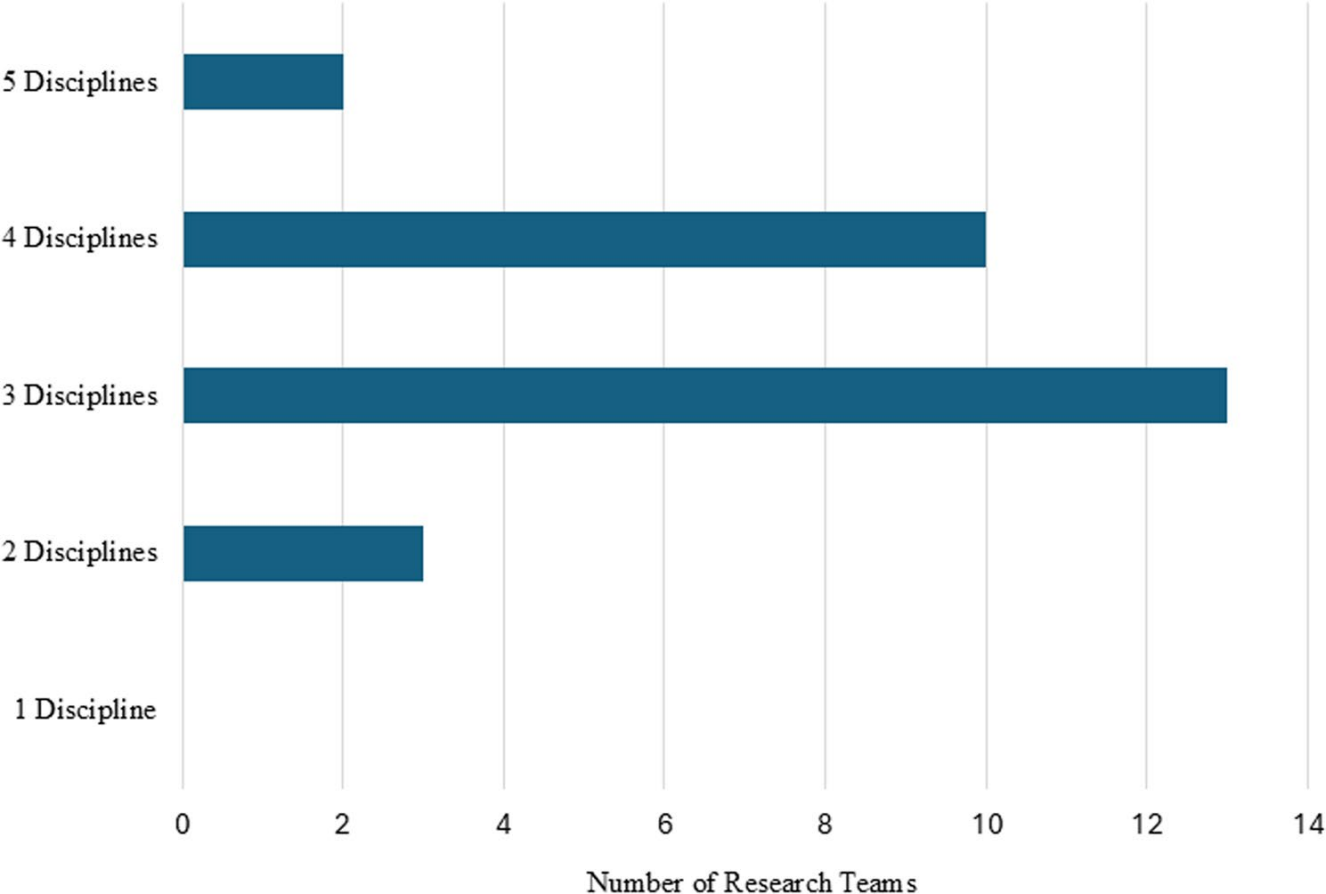
Number of disciplines represented on 28 CIRHT-UM seed grant research teams

Overall, most participants had limited prior experience with interdisciplinary research collaboration (Table 2). The majority of participants included three different professional disciplines on their CIRHT-UM seed grant research team (Fig. 2). There were no demographic or professional differences between the focus group and individual interview participants. Both participant groups were similar in gender, professional mix, interdisciplinarity, and prior research experience. At the time of the interviews, 26 of the 28 participants had completed their protocols and data analysis, and were writing manuscripts.

**Table 2:** Prior experience and disciplines on research teams of 28 Zambian CIRHT-UM seed grant awardees

Prior experience in interdisciplinary, collaborative research	*n* (%)
No prior experience	13 (46%)
Minimal experience (1–2 projects)	10 (36%)
Moderate experience (3–5 projects)	5 (18%)
Extensive experience (> 5 projects)	0 (0%)

**Fig. 3:**
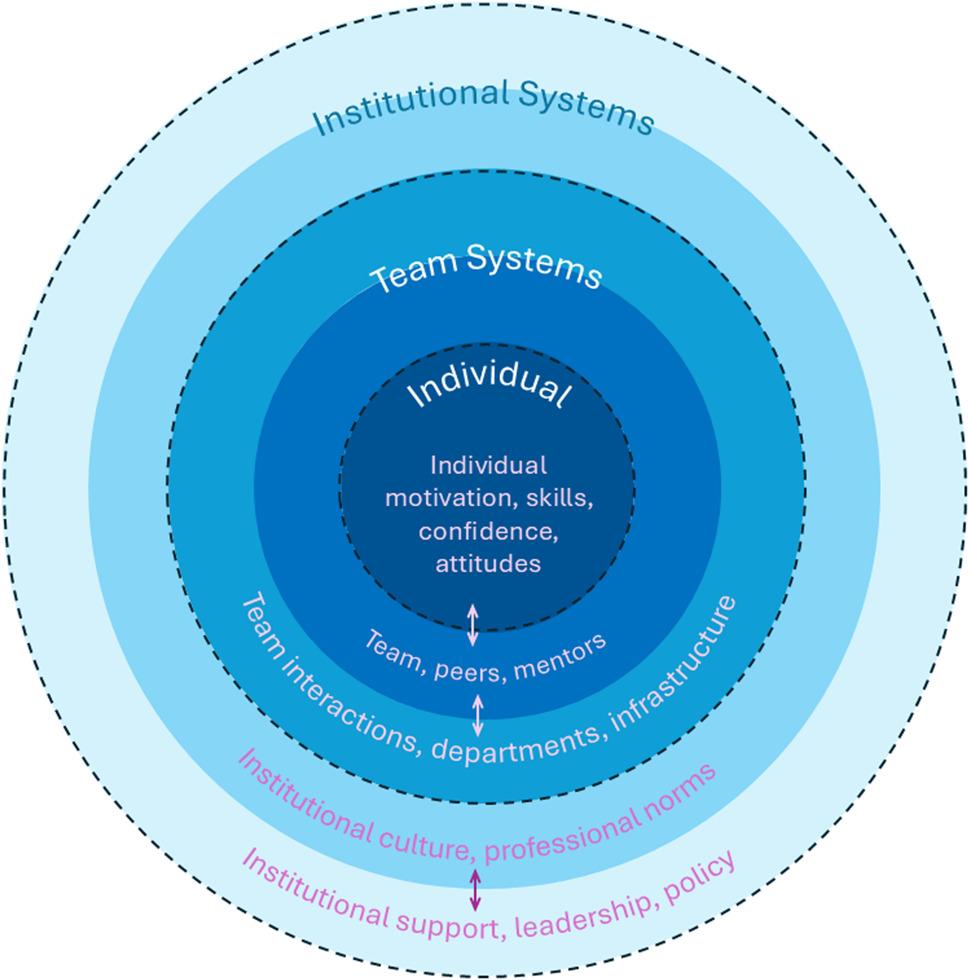
Integrated Ecological and Readiness Framework for Collaborative Skills Development

Our qualitative study explored the experiences, perceptions, and outcomes related to collaborative skills development during the CIRHT-UM program, drawing on Bronfenbrenner’s *Ecological Systems Theory* to position findings within multilayered ecological contexts, and using the *Gearing Up* framework to interrogate individual and systemic readiness for collaboration. Four interrelated themes were generated from the data, each spanning multiple ecological layers and reflecting varying degrees of readiness, capacity, and influences on collaboration. Rather than centering our analytic process on reaching saturation, we focused on capturing and representing the complexity and range of perspectives shared by all participants. This approach provided a multidimensional understanding of collaborative skill development across varied professional backgrounds and contexts. We present our findings under the following four key themes: (1) Expansion of professional networks and interdisciplinary exposure; (2) Barriers created by hierarchies, siloed work, and inequities; (3) Growth in communication, leadership, and problem-solving skills; and (4) Influence of environmental constraints and systemic supports. Figure 3 shows our *Integrated Ecological and Readiness Framework for Collaborative Skills Development*.

This diagram presents Bronfenbrenner’s ecological systems as shaded nested circles representing levels of influence on collaborative research skill, ranging from the individual to broader societal structures. At each layer, key domains of readiness from the *Gearing Up* framework (e.g., individual motivation, team capacity, institutional support) reflect the specific readiness and capacity needed at individual, team, and institutional levels for collaboration. To clarify how our themes relate to ecological levels and readiness factors, we included Table 3, an integrated schematic showing examples of how each theme mapped onto Bronfenbrenner’s nested systems and the *Gearing Up* readiness domains. For instance, communication skill growth was situated within the microsystem and linked to individual readiness, while institutional barriers like workload mapped to the exosystem and organizational readiness.

**Table 3:** Mapping of themes to Bronfenbrenner’s *Ecological Systems Theory* levels and *Gearing Up* readiness factors

Theme	Bronfenbrenner Ecological Systems Theory level(s)	Gearing Up readiness domain(s)	Illustrative example
Expansion of professional networks and interdisciplinary exposure	Mesosystem, Macrosystem	Team capacity, Organizational support	Cross-disciplinary workshops, network growth
Barriers: institutional hierarchies, siloed work, inequities	Exosystem, Macrosystem	Organizational readiness	Workload pressures, hierarchical culture
Growth in communication, leadership, and problem-solving skills	Microsystem, Mesosystem	Individual readiness, Team capacity	Improved confidence, peer learning
Influence of workload, mentorship, and systemic supports	Exosystem, Mesosystem	Organizational support, Team capacity	Mentor availability, time constraints

*Legend*: Bronfenbrenner *Ecological Systems Theory* level(s) include Microsystem: Individual-level interactions; Mesosystem: Relationships among immediate environments (teams); Exosystem: Institutional structures and policies; Macrosystem: Societal cultural norms. *Gearing Up* readiness domains: Individual readiness: Motivation, skill, preparedness; Team capacity: Functioning, knowledge-sharing; Organizational support: Policies, resources

### Expansion of professional networks and interdisciplinary exposure

Across focus groups and interviews, participants described the CIRHT-UM program as a catalyst for interdisciplinary exposure, echoing Bronfenbrenner’s mesosystem. Many participants, especially those with little prior collaborative research experience, highlighted how the program provided structured opportunities to interact with professionals from different disciplines such as nursing, medicine, midwifery, public health, and social sciences. As one nursing lecturer mentioned in an IDI, *“For me*,* being on this project kind of provided a platform for me to venture into my own research… and the interaction with people from other disciplines*,* I came to know a lot*,* especially those things I didn’t know before.”* (Bronfenbrenner mesosystem/macrosystem: exposure; *Gearing Up*: capacity strengthening).

The design of the CIRHT-UM training program, which explicitly encouraged multi-disciplinary teams, was credited with enlarging professional networks and cultivating an appreciation for the diverse expertise required for robust research. The *Gearing Up* lens generated that while the program’s structure fostered exposure and initial networking (organizational readiness), the capacity for deeper, sustained interdisciplinary work depended on both individual openness and institutional support mechanisms. As one physician noted in a focus group, *“It was very motivating*,* looking at the sponsorship and also looking at the various collaborators. They were very supportive in encouraging us to work on the project*,* and we were taken through every step.”* (*Gearing Up* organizational/team readiness: support structures, mentorship, resources).

### Barriers created by hierarchies, siloed work, and inequities

Despite the intended collaborative spirit, participants cited persistent barriers at multiple levels, such as professional hierarchies, siloed working styles, and unequal perceived value among disciplines, that stymied initial interdisciplinary collaboration. Some participants described a disparity between theoretical support for teamwork and the practical reality, where individuals mostly remained “PIs of their own projects,” working alongside but not truly with others. According to a midwife in an IDI, *“The biggest barrier for interdisciplinary research is the fact that we have too many tasks in the schools*,* too much work*,* too many also deadlines… Our work is extremely*,* extremely demanding and it’s difficult to even now work in a collaborative team. I wish that we had time for research.”* (Bronfenbrenner exosystem: institutional constraints and time; macrosystem: work culture; *Gearing Up*: organizational readiness/timing gaps).

Hierarchies and competition occasionally led to diminished collaboration, with more dominant disciplines sometimes overshadowing others. The *Gearing Up* framework further illuminated systemic barriers, such as insufficient availability of some mentors and limited protected time for team-based work, highlighting gaps in organizational capacity and preparedness that hampered cross-disciplinary engagement. As a public health lecturer mentioned in an FGD, *“Everyone was a PI with their own project. That meant collaboration was limited because you had to focus on your own deliverables*,* and so team meetings or joint work were rare.”* (*Gearing Up*: individual and organizational readiness not aligned for team collaboration).

### Growth in communication, leadership, and problem-solving skills

Participants commonly reported personal and interpersonal growth readiness, including enhanced communication, problem-solving, and leadership skills. They expressed feeling prepared and willing to develop better skills for interacting and collaborating effectively with others. Many described a transformation from initial uncertainty or reticence to increasingly confident participation in group meetings, trainings, and peer review processes during the course of the program. A midwifery lecturer in an FGD said, *“I learnt to be patient… There are times when I wanted things to work my way*,* but the CIRHT project taught me to be patient with people and processes*,* to communicate better and to adjust.”* (*Gearing Up* individual readiness/skills: communication, problem-solving). These developments were attributed to repeated exposure to group-based research trainings, the presence and encouragement of experienced mentors, and constant feedback from a diverse set of peers. A public health lecturer in an IDI noted, *“Leadership was more than just leading my study. It was also being a team player*,* offering help to other people’s projects*,* contributing and learning—being able to contribute depending on the skills I have.”* (Bronfenbrenner: micro/mesosystem; *Gearing Up*: leadership/team readiness).

Individual-level readiness (*Gearing Up*) grew over the program as participants navigated ecological settings that encouraged sharing, questioning, and decision-making across disciplinary lines. Notably, some participants reported that their increased confidence in communicating and leading within teams translated into their broader professional practice including teaching students and managing clinical teams. According to a public health lecturer in an FGD, *“I saw myself exercising almost all the roles of leadership… I used listening skills*,* communicated*,* and also exercised a lot of patience*,* especially when working with vulnerable populations and team members experiencing issues. Sometimes you’re a counselor*,* sometimes a mentor. So many leadership skills at play.”* (*Gearing Up* individual readiness: confidence, networking).

### Environmental constraints and systemic supports

Environmental and institutional contexts profoundly influenced collaborative outcomes. Scheduling conflicts, lack of protected time for collaboration, and competing work responsibilities, features of Bronfenbrenner’s exosystem and macrosystem, were frequently cited as practical barriers to deeper teamwork. As a midwifery lecturer in an FGD noted, *“Workload is huge in our school… you literally do not knock off*,* and sometimes you would want to do a good job but fail to fulfill every aspect where you are needed for collaborative work.”* (Bronfenbrenner exosystem: institutional workload).

At the same time, participants emphasized systemic support, such as well-run training sessions, clear communication channels (e.g., WhatsApp groups), supportive mentorship, and access to funding enabled progress despite these barriers. For example, a nursing lecturer in an FGD mentioned, “*In my team*,* after realizing some were very quantitative and some qualitative*,* we divided up tasks*,* and people took the lead on what they knew best. I learnt a lot about qualitative from one team member who taught us and even gave assignments.”* (Bronfenbrenner mesosystem, interactions between people; *Gearing Up*: peer learning, skills transfer) Suggestions for programmatic improvement focused on the need for additional training, more flexible schedules, and additional protected time to ensure ongoing interdisciplinary engagement, indicating a keen awareness of the need for both individual capability and organizational readiness as described in the *Gearing Up* framework.

### Comparison of focus group and individual interview findings

The complementary use of FGDs and IDIs offered multi-layered insights that align with both Bronfenbrenner’s *Ecological Systems Theory* and the *Gearing Up* framework. FGDs predominantly illuminated the collective and organizational dynamics at the meso- and exosystem levels, revealing how team processes, shared norms, group learning, and institutional support or barriers influenced participants’ readiness and capability for collaboration. Participants frequently reached consensus on common challenges and strengths, providing a systemic view of the collaborative environment and illustrating how group interactions and organizational context shaped their collective experience, consistent with both the mesosystem and the organizational readiness aspects of the *Gearing Up* framework.

Individual interviews, in contrast, detailed the microsystem by capturing individualized narratives, offering deeper insight into intrinsic motivation, personal growth trajectories, and unique strategies for overcoming barriers. These interviews spotlighted how individuals perceived their development, the personal impact of the program, and factors at the individual level that influenced readiness and capability to collaborate. The IDIs highlighted aspects of participant agency and reflexivity, echoing the *Gearing Up* framework’s dimension of individual readiness and capability, as well as Bronfenbrenner’s focus on the central role of personal context within the wider system.

The complementary use of FGDs and IDIs generated multi-layered insights. FGDs predominantly illuminated collective and organizational dynamics, revealing how team processes, shared norms, group learning, and institutional context shaped participants’ readiness and capacity for collaboration. In contrast, IDIs provided deeper insight into personal growth trajectories, intrinsic motivation, and individualized strategies, highlighting diversity in readiness and unique responses to common barriers. Integrating findings from both methods enabled a robust analytical framework that mapped experiences across ecological and readiness levels.

## Discussion

This study explored participants’ experiences, perceptions, and outcomes related to collaborative skills development within a health research capacity strengthening program in Zambia. Drawing on Bronfenbrenner’s *Ecological Systems Theory* and the *Gearing Up* framework, we positioned our findings within a comprehensive view of ecological and readiness-based influences on teamwork and interdisciplinary capacity. Participants reported significant gains in teamwork, leadership, and communication, while also highlighting persistent structural challenges, such as entrenched hierarchies, heavy workloads, and inconsistent institutional support. These results reinforce that effective interdisciplinary collaboration requires both individual readiness and robust environmental and organizational backing.

Our findings are consistent with previous research highlighting the importance of flattening hierarchies and providing structured mentorship to enhance teamwork and innovation in health research settings [32, 33]. As with studies in other sub-Saharan African contexts [34, 35], we found that institutional silos and workload pressure undermined collaborative efforts, despite well-designed program structures. Our findings regarding the profound impact of peer learning and informal cross-disciplinary networks align with the work of Amegee Quach et al. [36], who found that learn-by-doing approaches and relationship-building often foster professional trust, confidence, and innovation in global health research partnerships. Finally, the importance participants placed on institutional resources, specifically regular meetings and communication tools, reflects findings by Simons and colleagues whose review showed that structured collaboration mechanisms, such as scheduled interactions and effective communication platforms, are key components of successful interdisciplinary and inter-organizational efforts in health and social care [37].

Methodologically, utilizing both FGDs and IDIs enabled us to map both collective team dynamics and individualized growth trajectories. Focus groups captured the broader program climate and systemic factors, while individual interviews uncovered unique personal experiences, strategies, and growth. Integrating the Bronfenbrenner and *Gearing Up* frameworks allowed us to trace how support and barriers at each ecological level shaped collaborative readiness and performance.

### Implications for research programs and institutional policy

Our findings have several implications that lead to recommendations for research training program design and institutional policy. Capacity-strengthening efforts need to address both individual readiness (e.g., communication and leadership training) and institutional support (e.g., university/departmental policies, flexible scheduling), operationalizing readiness at multiple ecological levels. At the individual level, programs could incorporate explicit skills training in communication, conflict management, and leadership development, while also fostering reflexivity about disciplinary identities and readiness for collaboration [38, 39]. At the institutional level, securing leadership commitment to team science is essential and has potential to be achieved through policy alignment, formal recognition of collaborative activities, and appropriate resource allocation [40, 41]. Establishing protected time and flexible scheduling for collaborative work could facilitate greater participation and sustained engagement [39, 42].

At the policy level, it is important to advocate for explicit policies that protect and incentivize collaboration, covering areas such as resource mobilization and the establishment of formalized networking structures [43, 44]. Equipping researchers to recognize and navigate institutional and structural barriers, encouraging reflexivity about their own disciplinary identities and assumptions, could strengthen collective research capacity and foster a genuinely collaborative scientific culture [45, 46]. Taken together, these implications suggest that building collaborative skills requires coordinated efforts at both the individual and institutional levels, emphasizing structural change, explicit training objectives, and ongoing support mechanisms to ensure lasting impact.

Program designers could embed modules on communication, teamwork, conflict management, and leadership into research training, creating structured opportunities for practice and reflection [47]. Establishing multidisciplinary teams through intentional matching and cross-disciplinary workshops, alongside structured mentorship that fosters inclusive environments, could help break down hierarchical barriers [48]. Institutions can support collaboration by formally recognizing team science in advancement criteria, providing protected time and flexible scheduling, and maintaining regular communication platforms for ongoing peer learning [49]. Policymakers could invest in interdisciplinary projects, mentorship programs, and collaborative workspaces, and promote ethics and funding guidelines that encourage cross-sector partnerships, while targeting policies to reduce hierarchies and workload imbalances [50]. Collectively, these strategies could build collaborative skills, strengthen team effectiveness, and improve research and health outcomes.

### Strengths and limitations

Our study offers several new contributions to the literature on collaborative skills development in health research training. Our results add new evidence to the pivotal role of egalitarian team environments and peer learning in building confidence and research competencies. Our context-specific findings from Zambia add much-needed qualitative evidence from sub-Saharan Africa, illuminating how entrenched hierarchies, workload pressures, and institutional silos can persist as barriers, even within well-structured interdisciplinary programs. While such challenges are prevalent in academic institutions across the globe, resource-limited settings may have less flexibility to address these barriers.

Several features strengthen the validity and transferability of our findings, including the use of both focus groups and individual interviews to capture diverse perspectives, purposive sampling across key health professions and career stages, and rigorous analytic procedures. We acknowledge that power dynamics, as all participants were CIRHT-UM seed grant recipients, may have influenced willingness to participate and candor during interviews and focus groups. Although independent interviewers and assurances of confidentiality aimed to minimize bias, some social desirability or perceived obligation may remain, affecting interpretation of findings. Additionally, the small number of interviews and focus groups may have limited the range of perspectives and the transferability of results; future studies with larger, more varied samples could strengthen generalizability. Throughout our data analysis, we found that participant accounts were largely consistent with respect to collaborative skill development, and did not encounter substantial deviant or contradictory cases.

### Directions for future research

Building on our findings, future studies should consider longitudinal designs to examine the persistence of collaborative skills and impacts on research productivity and outcomes. Comparative studies across different regional or institutional contexts would help clarify how local culture, policy, and resource availability influence the effectiveness of collaboration-focused interventions. Further work is also needed to develop and test targeted strategies for overcoming persistent barriers, such as entrenched hierarchies and institutional silos, particularly in low-resource and high-workload environments.

## Conclusion

Our study underscores the critical importance of addressing both individual and systemic factors to foster effective interdisciplinary collaboration in health research. By highlighting key barriers and facilitators, we recommend that training programs incorporate explicit, multi-level strategies, such as protected time and structured team-based learning, to strengthen collaborative capacity. Implementing these changes will better prepare health researchers to meet the complex, team-oriented demands of contemporary science and ultimately strengthen research capacity and impact in similar settings.

While our findings are relevant to countries with similar resource and institutional challenges, certain contextual factors require consideration. Barriers like hierarchies, limited protected research time, and workload pressures are common and make structured mentorship, team-based learning, and institutional support broadly applicable. However, features such as the unique professional dynamics in Zambian health institutions and the specific CIRHT-UM program design may limit direct transferability. Strategies should therefore be adapted to fit local cultures, resources, and policies, with future programs assessing context-specific factors for optimal impact.

## Supplementary Information

Supplementary Material 1.

Supplementary Material 2.

Supplementary Material 3.

## Data Availability

The datasets used and/or analysed during the current study are available from the corresponding author on reasonable request.

## Abbreviations

CIRHT-UM: Center for International Reproductive Health Training at the University of Michigan
COREQ: Consolidated Criteria for Reporting Qualitative Research
IDIs: in-depth interviews
IRB: Institutional Review Board
FGDs: focus group discussions
MMed: Master of Medicine
MPH: master’s in public health
PI: principal investigator
SRHR: sexual and reproductive health and rights
UNZA: University of Zambia
