# Supplementary material for: Interdisciplinary collaborative skill development in a health research training program in Zambia
Source: BMC Med Educ. 2026 Feb 21;26:512. doi: 10.1186/s12909-026-08867-8 (PMC13032487; doi:10.1186/s12909-026-08867-8)
Supplement: Supplementary file 1 — Supplementary Material 1. [file 12909_2026_8867_MOESM1_ESM.docx]

**Appendix 1: Interview guide questions for focus groups and individual interviews for collaborative research skills development study in Zambia**

1. How have you experienced interdisciplinary collaboration in your previous work before joining the CIRHT research training program?
2. At the start of the CIRHT training program, did you have any expectations about  collaborative skills development and if so what were they?
3. Did the research training program influence your ability to work effectively as part of a team and in what ways?
4. If the research training program did not influence your ability to work effectively within a team, where do you think the training fell short?
5. Did the program facilitate your growth? If so, can you share specific experiences from the CIRHT program that facilitated your growth in communication within an interdisciplinary research team?
6. Did you notice any changes in your leadership skills during the program? If so, what leadership skills do you feel you developed during the program, and how were these skills fostered?
7. Which components of the CIRHT program did you find most effective for developing your collaborative skills, and why?
8. Which components of the CIRHT program were least effective?
9. From your experiences, share with us one event during your research program where you observed effective interdisciplinary collaboration? What made it effective?
10. Have you applied skills learned in the CIRHT program to address conflict resolution? If so,  can you share an example?
11. Are there still areas that you need to further develop in the area of conflict resolution?
12. What do you think hinders interdisciplinary research in your setting? What barriers can researchers come across?
13. Please share with us one event during your research program where you observed ineffective interdisciplinary collaboration? What made it ineffective?
14. What strategies have you found effective in overcoming difficulties when collaborating with diverse research teams?
15. Has your perspective on the importance of collaborative skills in research evolved throughout your experience in the CIRHT program? If yes, why.
16. What skills do you want to continue developing to advance your interdisciplinary collaboration?
17. If you could suggest any changes or additions to the CIRHT program to enhance interdisciplinary collaborative skill development, what would they be?
18. Is there anything else you would like to share about your collaborative experiences with the CIRHT seed grant?
