# Supplementary material for: Interdisciplinary collaborative skill development in a health research training program in Zambia
Source: BMC Med Educ. 2026 Feb 21;26:512. doi: 10.1186/s12909-026-08867-8 (PMC13032487; doi:10.1186/s12909-026-08867-8)
Supplement: Supplementary file 2 — Supplementary Material 2. [file 12909_2026_8867_MOESM2_ESM.docx]

**Appendix 2: Code book for collaborative research skills development study in Zambia**

| **Initial Code** | **Definition** |
| --- | --- |
| Interdisciplinary Exposure (Pre-Program) | Participation in interdisciplinary projects or teams before CIRHT |
| Previous Barriers | Experienced obstacles to collaboration due to workplace culture, structure, or policies |
| Early Positive Collaborations | Beneficial joint projects or teamwork before CIRHT |
| Anticipated Skills or Knowledge | Skills/knowledge participants specifically hoped to gain (e.g., data analysis, proposal writing) |
| Surprises and Exceeded Expectations | Participant describes learning or benefit that surpassed original hopes or assumptions |
| Absence of Expectations | Stated absence or minimal expectations before joining program |
| Expectations Met/Unmet | Explicit indication of whether original expectations were fulfilled or disappointed |
| Professionally Egalitarian Environments | Teamwork characterized by true equality among professional roles/cadres/disciplines |
| Positive Emotional Response to Equality | Statements of comfort, satisfaction, or inclusion due to flattened hierarchy |
| Peer-to-Peer Learning | Direct learning from fellow participants (not from assigned mentors or experts) |
| Mentoring by Supervisors or Experts | Receiving guidance or targeted skill-building from designated supervisors or subject experts |
| Learning from Colleagues | Any learning gained by working closely with colleagues in group or project |
| Skill Use in Team Settings | Use of collaboration, leadership, or teamwork skills during research and group work |
| Enhanced Research Outputs or Practice | Improved research design/outcomes, confidence, or new ways of working as a result of collaboration |
| Distribution of Roles (Skill-building Activities) | Assignment and experience of specific project roles and corresponding learning activities |
| Structural Enablers (Scheduled Meetings, Communication Tools) | Program features (e.g., regular meetings, reminders, use of WhatsApp) that facilitate collaboration |
| Design Barriers | Aspects of team structure or organization that hindered effective collaboration |
| Multitasking and Schedule Conflicts | Struggles to juggle many concurrent responsibilities or priorities |
| Team Availability/Set Timings | Difficulty finding meeting times or synchronizing work with group members |
| Missed Opportunities Due to Workload/Schedule | Skipped activities/mentoring due to workload, leading to unfulfilled learning or collaboration |
| Enhanced Group Communication | Improved quality, clarity, or frequency of communication among team members |
| Problematic Team Interactions/Managing Expectations | Any experiences of frustration, delay, or need for conflict resolution in group work or leadership tasks |
| Team Building/Coordination | Experience of organizing, managing, setting up, or facilitating a team or sub-team |
| Time/People Management | Overseeing, scheduling, and motivating people in a research/project context |
| Record Keeping/Accountability | Tracking and documenting research processes, approvals, and decisions as a leadership responsibility |
| Patience/Compassion/Counselling | Exercising patience, empathy, and supportive communication with team members (including in conflict) |
| Representation & Inclusion | Efforts to ensure team composition is inclusive or represents diverse perspectives/groups |
| Institutional Changes | Suggestions for changes in policy or institutional approaches to improve collaboration |
| CIRHT Programmatic Changes | Suggestions for changes in CIRHT program structure |

**Collapsed Codes**

| **Code** | **Definition of Code** |
| --- | --- |
| Prior Experiences with Collaboration | Experiences with interdisciplinary or team-based research activities prior to the CIRHT program. |
| Expectations of Collaborative Skills Development | Participants' pre-program expectations/assumptions about team research and skill training. |
| Inclusiveness / Dissolved Hierarchies | Experiences of teamwork marked by professional equality and absence of traditional hierarchies. |
| Peer Learning, Mentorship, and Networking | Informal or formal mentorship, peer support, and networking experienced in the program. |
| Application and Impact of Collaborative Skills | Real-world use of collaborative skills; changes in practice, teamwork, or personal growth. |
| Program Design—Team Structure, Roles, and Inclusion | Programmatic/structural factors shaping the collaboration experience. |
| Challenges of Competing Responsibilities/Time Constraints | Time, workload, and scheduling as practical barriers to collaboration. |
| Communication, Conflict Management, and Team Dynamics | Experiences with communication, conflict, and team dynamics in collaborative settings. |
| Leadership Skills Developed/Practiced | Growth in leadership ability through team/project work. |
| Recommendations/Suggestions for Improvement | Participant-generated ideas for improving future programs or teamwork. |

**Mapping of Themes**

| **Collapsed Code** | **Theme** |
| --- | --- |
| Prior Experiences with Collaboration | Expansion of Professional Networks and Interdisciplinary Exposure |
| Expectations of Collaborative Skills Development | Expansion of Professional Networks and Interdisciplinary Exposure |
| Inclusiveness / Dissolved Hierarchies | Expansion of Professional Networks and Interdisciplinary Exposure |
| Peer Learning, Mentorship, and Networking | Expansion of Professional Networks and Interdisciplinary Exposure;  Growth in Communication, Leadership, and Problem-Solving Skills |
| Application and Impact of Collaborative Skills | Application, Impact, and Challenges of Collaborative Skills in Practice |
| Program Design—Team Structure, Roles, and Inclusion | Expansion of Professional Networks and Interdisciplinary Exposure;  Environmental Constraints and Systemic Supports |
| Inclusiveness / Dissolved Hierarchies | Barriers Created by Hierarchies, Siloed Work, and Inequities |
| Challenges of Competing Responsibilities/Time Constraints | Barriers Created by Hierarchies, Siloed Work, and Inequities |
| Communication, Conflict Management, and Team Dynamics | Growth in Communication, Leadership, and Problem-Solving Skills |
| Leadership Skills Developed/Practiced | Growth in Communication, Leadership, and Problem-Solving Skills |
| Recommendations/Suggestions for Improvement | Environmental Constraints and Systemic Supports |
